# Editorial: Chronic Inflammation and Neurodegeneration in Retinal Disease, Volume II

**DOI:** 10.3389/fphar.2022.915960

**Published:** 2022-05-31

**Authors:** Claudio Bucolo, Julie Sanderson, Settimio Rossi

**Affiliations:** ^1^ Department of Biomedical and Biotechnological Sciences, School of Medicine, University of Catania, Catania, Italy; ^2^ University of East Anglia, School of Pharmacy, Faculty of Science, Norwich, United Kingdom; ^3^ Multidisciplinary Department of Medical, Surgical and Dental Sciences, University of Campania “Luigi Vanvitelli”, Naples, Italy

**Keywords:** inflammation, retinal diseases, diabetic retinopathy, age-related macular degeneration, retinitis pigmentosa

Inflammation and neurodegeneration have a widely recognized role in the pathogenesis of the main retinal conditions. However, the exact mechanism through which inflammation causes alteration of the retinal structure—with consequent dysfunction of the retinal pigment epithelium, of neurons, and ultimately of photoreceptors—is not entirely known. This Research Topic “*Chronic Inflammation and Neurodegeneration in Retinal Disease, Volume II*” presents eight original research articles and one mini review from seven different countries with important contributions in the field of retinal inflammation. Most of the contributions are related to diabetic retinopathy (DR), the others cover age-related macular degeneration (AMD), retinitis pigmentosa, and the spontaneous polygenic model of inherited retinal dystrophy.

The review by Stravalaci et al. focused on long pentraxin 3 (PTX3), an emerging new player in ocular homeostasis and a potential pharmacological target in neurodegenerative disorders of the retina. Physiologically present in the human eye and induced in inflammatory conditions, this protein is strategically positioned at the blood retinal barrier interface, where it acts as a “molecular trap” for complement and modulates inflammation both in homeostatic and pathological conditions such as AMD and DR. Gesualdo et al. presented an interesting study on fingolimod and DR, investigating the interactions between fingolimod, a sphingosine 1-phosphate receptor (S1PR) agonist, and melanocortin receptors 1 and 5 (MCR1, MCR5). This Research Topic is a typical example of repurposing since fingolimod is a drug approved to treat relapsing-remitting multiple sclerosis. The authors demonstrated, in an *in vivo* model of DR, that fingolimod has anti-angiogenic activity mediated not only through S1P1R, but also by melanocortin receptors. Another interesting pre-clinical study on DR was provided by Canovai et al. The authors showed the efficacy of a novel substance containing cyanidin-3-glucoside (C3G), verbascoside, and zinc to maintain the integrity of the blood retinal barrier and retinal function in streptozotocin-induced diabetic rats.

Accumulating data provide evidence for a pivotal role of Müller cells in the pathogenesis of DR. In this regard the *in vitro* study by Schmalen et al. underlined the importance of Müller cell signaling in the inflamed retina, indicating an active role in chronic retinal inflammation. These authors demonstrated an intense signaling capacity of Müller cells, which reacted in a highly discriminating manner upon treatment with different cytokines, showing several characteristics of atypical antigen-presenting cells.

An important clinical contribution on the Research Topic of DR has been provided by Parravano et al., where the authors carried out a randomized clinical trial on patients with diabetic macular edema (DME) treated with a special oral curcumin formulation with a polyvinylpyrrolidone-hydrophilic carrier and intravitreal injections of dexamethasone. They demonstrated a significant reduction in central retinal and inner retinal layer thickness with the combined therapy in patients affected by DME. This study also showed that the pharmacological combination therapy (curcumin and dexamethasone) was well-tolerated. On this regard, a good long-term safety profile of intravitreal dexamethasone has been demonstrated on real-world studies when used to manage DME (Bucolo et al., 2018). Further, these findings are in line with recent literature data that showed retinal protective effects by curcumin against high glucose damage (Bucolo et al., 2019). Another important contribution on the Research Topic of DME is the bibliometric study and visualization analysis by Lin et al.. They used CiteSpace and VOSviewer software to evaluate the Web of Science Core Collection publications and to build visualizing maps to describe the research progress on the use of steroids to treat DME. They concluded that while anti-VEGF therapy is the first-line treatment for DME and retinal vein occlusion (RVO)-induced macular edema, steroid implant is a valid option for DME patients not responding to anti-VEGF therapy and non-DME patients with macular edema. The final contribution on the topic of DME focused on the anti-inflammatory effects of subthreshold micropulse yellow laser (SMYL). The authors, Bonfiglio et al., demonstrated that SMYL may reduce macular thickening and improve best-corrected visual acuity in eyes with persistent macular edema after pars plana vitrectomy and membrane peeling for tractional DME.

A group of scientists from University of Tennessee (Hollingsworth et al.) have used systems genetics to identify possible models of spontaneous polygenic AMD by mining the BXD family of mice using single nucleotide polymorphism analyses of known genes associated with the human retinal disease. The goal of these scientists was to propose a pre-clinical mouse model (BXD32) to better understand the pathophysiology of progressive retinal dystrophies and discover efficacious treatments. Their study demonstrated that the BXD32 mouse strain exhibits a severe neurodegenerative phenotype accompanied by adverse effects on the retinal vasculature. Finally, Canto et al. showed that sulforaphane, a natural compound, modulates the inflammation and delays neurodegeneration in a retinitis pigmentosa (RP) mouse model. Specifically, they assessed the modulation of glial cells in the RP rd10 mouse model showing that sulforaphane treatment regulated the microglial activation state.

Overall, the contributions of the present Topic Research focused on inflammation and retinal degeneration, highlighting new insights in retinal diseases mechanisms and novel pharmacological approaches.
